# Inter-Examiner and Intra-Examiner Reliability of Quantitative and Qualitative Ultrasonography Assessment of Peripheral and Respiratory Muscles in Critically Ill Patients

**DOI:** 10.3390/ijerph20095636

**Published:** 2023-04-25

**Authors:** Felipe Douglas Silva Barbosa, José Lucas Dos Santos, Maria Emilia Dantas Alves, Juliana de Ávila Barreto Alves, Telma Cristina Fontes Cerqueira, Valter Joviniano De Santana Filho

**Affiliations:** 1 Graduate Program in Health Sciences, Federal University of Sergipe, Aracaju 49060-100, Brazil; vjsf@academico.ufs.br; 2 Family Health and Occupational Therapy Department, Faculty of Medicine, Federal University of Bahia, Salvador 40026-010, Brazil; 3 University Hospital of Lagarto, Federal University of Sergipe, Lagarto 49400-000, Brazil; jose.lucas@ebserh.gov.br (J.L.D.S.); juliana.avila@ebserh.gov.br (J.d.Á.B.A.); 4 Multiprofessional Integrated Residency Program in Hospital Care, University Hospital of Lagarto, Federal University of Sergipe, Lagarto 49400-000, Brazil; milinha_d@academico.ufs.br; 5 Department of Physiotherapy of Lagarto, Federal University of Sergipe, Lagarto 49400-000, Brazil; telmac@gmail.com; 6 Department of Physiotherapy, Federal University of Sergipe, São Cristovão 49100-000, Brazil

**Keywords:** intensive care unit, ultrasonography, muscles, test reproducibility

## Abstract

ICU patients are exposed to several factors that can lead to muscle structural and functional changes, and ultrasonography can identify them. Although several studies have analyzed the reliability of muscle ultrasonography assessment, a protocol with more muscle assessments becomes a challenge. The aim of this study was to analyze the inter and intra-examiner reliability of peripheral and respiratory muscle ultrasonography assessment in critically ill patients. The sample size was 10 individuals aged ≥ 18 years who were admitted to the ICU. Practical training of four health professionals from different backgrounds was performed. After training, each examiner acquired three images to assess the thickness and echogenicity of the muscle groups: biceps brachii, forearm flexor group, quadriceps femoris, tibialis anterior and diaphragm. For the reliability analysis, an intraclass correlation coefficient was performed. Six hundred US images were analyzed for muscle thickness and 150 for echogenicity. Excellent intra-examiner reliability for echogenicity (ICC: 0.867–0.973) and inter-examiner reliability for thickness were found in all muscle groups (ICC: 0.778–0.942). For muscle thickness intra-examiner reliability, excellent results were found (ICC: 0.798–0.988), with a “good” correlation in one diaphragm assessment (ICC: 0.718). Excellent inter- and intra-examiner reliability of the thickness assessment and intra-examiner echogenicity of all muscles analyzed were found.

## 1. Introduction

Individuals hospitalized in Intensive Care Units (ICU) are exposed to several factors that can lead to structural and functional changes in the muscles, which may be related to important clinical outcomes, such as functional disability and mortality [1]. These patients can lose about 2% of muscle mass per day, with a higher loss during the first 7–10 days of immobility [2]. Moreover, immobility in bed for more than 72 h can cause damage up to 5 years after hospital discharge [3]. Furthermore, the deleterious effects suffered by the musculoskeletal system are generalized during ICU stay, affecting both peripheral and respiratory muscles [4].

According to Tillquist et al. [5], a good evaluation in the ICU is important for the early identification of impairments and to guide the treatment accordingly. Among these methods, ultrasonography (US) has been identified by The European Working Group on Sarcopenia as a useful method for evaluating skeletal muscle, being able to identify structural, quantitative and qualitative changes in peripheral and respiratory muscles, in addition to considering its characteristics of being an accessible tool at the bedside, non-invasive and portable [6], although accepting that further research is required [7,8].

The US diagnostic accuracy to verify muscle changes in the ICU has been analyzed in several studies, proving that this evaluation method is capable of verifying changes in muscle thickness, cross-sectional area and echogenicity [9,10,11]. Studies have shown that changes in muscle mass and quality may be associated with changes in strength and functional capacity. These studies diversely report the reduction in muscle thickness and cross-sectional area and increased echogenicity are associated with a decrease of muscle strength and functional capacity during the awakening of critically ill patients, mainly analyzing the quadriceps femoris and diaphragm and, less frequently, the upper limb muscles [12,13,14,15,16]. Considering this, ultrasonographic assessment of the muscle changes is suggested as a prognostic marker, while the patient cannot cooperate with volitional functional tests [11].

Furthermore, considering the different methodologies and results found, in addition to the fact that US is evaluator-dependent, in other words, dependent on the evaluator’s interpretation, inter- and intra-examiner reliability studies were performed. US has shown excellent inter-examiner and intra-examiner reliability, considering different levels of experience, settings, examiners' level of training and established protocols [5,17,18].

Even taking into consideration all the US potential and the results of the available studies, their interpretation and implementation are tough because of significant methodological weaknesses and lack of standardization of the ultrasonography methodology [19,20]. There are some concerns in regard to blinding assessment, appropriate training, reliability of landmarks, participant positioning, muscle assessment and so on [20]. Moreover, even though several studies have done the evaluation and analysis of different muscle groups, a huge amount of research has been focused on large muscle groups (e.g., quadriceps femoris), but it suggested that smaller muscles can also be interesting due to their specific function. Moreover, it is unclear if analyses of a muscle group, such as the quadriceps femoris, may represent the impairments related to the whole body, such as sarcopenia [7,8,21].

As studies have been conducted mainly considering usually one or two muscles per study, there is a lack of knowledge in regards to the reliability of the muscle assessment of the higher amount of muscles/muscles groups and a need for standardization of a protocol with a greater number of muscle assessments aiming to improve the ability to evaluate the global effects of muscle changes. As a first step, the analysis of the inter- and intra-examiner reliability of a protocol in this context is necessary, considering the complexity of the evaluation procedure. Thus, this research aims to analyze the inter-examiner and intra-examiner reliability of peripheral and respiratory muscle assessment through ultrasonography in critically ill patients. It was hypothesized that, after the training, examiners would have excellent inter and intra-examiner reliability in US muscle assessment.

## 2. Material and Methods

### 2.1. Study Design, Setting, and Ethical Considerations

This is an investigation of the inter- and intra-examiner reliability of muscle assessment through ultrasonography, which was conducted in a general ICU (medical and surgical) of a university hospital. This study followed the guidelines for reporting reliability and agreement studies (GRRAS) [22]. All subjects, or their guardians/family members/caregivers, who participated in this research were informed about the study conditions and signed the “informed consent form”. This study was approved by the Research Ethics Committee of the Federal University of Sergipe (approval number: 5.531.925).

### 2.2. Population, Sample, Inclusion, and Exclusion Criteria

The population of this study corresponds to individuals aged ≥ 18 years and who were admitted to the ICU. Participants were recruited by convenience, including participants who met the inclusion and exclusion criteria and were already admitted to the ICU, as well as participants who were admitted later until the required sample was reached.

The sample size was 10 participants. This value was determined from the recommendations of Walter et al. [23]. It included the acceptable reliability values (ICC = 0.70), the predicted reliability coefficient (ICC = 0.93, based on a previous study) [17], three repetitions of the task and levels of error type I (α = 0.05) and type II (β = 0.20). The result of the sample of nine individuals was obtained, but we recruited ten individuals to reduce losses.

Participants were included if they were ≥18 years old and were admitted to the ICU. Subjects were excluded if 1. they remained hospitalized for less than 48 h; 2. they had a pre-admission diagnosis of dementia; 3. they received home mechanical ventilation before admission; 4. they were diagnosed with terminal cancer or were in palliative care; 6. they had a second or subsequent admission to the ICU during the study period; or 7. they did not have family members or caregivers who could give information or sign the “informed consent form”.

### 2.3. Data Collection

Following the protocol described by Mayer et al. [18] approach, four health professionals (one nurse, two physiotherapists, and one occupational therapist) were trained by an expert Physiotherapist, Ph.D. and university lecturer, who had training and experience in muscle assessment through ultrasonography prior to implementation. Three of the examiners were well-trained and experienced, with more than 5 years of experience working in ICUs, with the exception of one physiotherapist who was ending her two-year residency program. All of them were from the research group and had experience with research projects. In regard to US muscle assessment, examiners had little to no prior experience with US muscle assessment, with one examiner (OT) with informal training and experience during the last year. During the data collection, they did not receive any other training.

Regarding the training program, face-to-face meetings were held, with a duration of about eight hours. Guidance was given regarding the ultrasonography handling, equipment regulation for the image acquisition (frequency, depth and gain), transducer positioning and the protocol to be used for participants' positioning, location of points of interest, identification of anatomical structures (subcutaneous tissue, fascia, muscle and bone) and acquisition of images, in addition to their storage. Moreover, it was provided training to acquire images and measure the thickness of all muscles/muscle groups analyzed in this research. A “guiding document” was available during the image acquisition, including the step-by-step to be performed and the points of interest.

After the training program, each of the 4 evaluators acquired three images to allow inter- and intra-examiner reliability analyses [12], which were obtained based on the structured protocol for subject positioning, determination of appropriate reference points and handling of the transducer, as reported.

To recruit participants, one of the four examiners contacted the prospective participants or their legal guardians in cases where the participant was unable to verify the inclusion and exclusion criteria and sign the informed consent form. The participants’ clinical information was collected after the completion of the US muscle assessment of all participants.

### 2.4. Ultrasonography Assessment Protocol

For image acquisition, a MindRay M30 portable ultrasound device (Shenzhen Mindray Bio-Medical Electronics Co., Ltd., Shenzhen, China) was used, with a model 75L38P linear transducer. The settings for image acquisition (frequency, depth and gain) were kept constant between examiners (MSK pre-set, frequency of 8.5 MHz, depth of 7.4 cm and gain of 64 db).

In regard to participants positioning in bed, all of them were examined in the supine position, in a semi-recumbent position with patient’s head of bed placed at 30-degree angle, with arms and legs extended and muscles in a relaxed state. All US images were obtained on the participant’s right side, independently and with alternation of evaluators for each image acquisition of all muscles, within 24 h after the first examiner’s evaluation to reduce fluctuations in measurement and analysis of muscle parameters.

After each image acquisition attempt, the subject was returned to its initial position, and the skin was cleaned to remove any gel or marks, ensuring the independent performance of image acquisition procedures. Independent positioning, landmark capture, and transducer handling techniques were emphasized to reduce the risk of measurement bias, such as anchoring. After each assessment, the ultrasonography device was reset to the home screen, with the new evaluator instructed to create a new file for subsequent image storage. This approach was used to minimize the risks of an examiner accessing images of the previous examiner. All images obtained by the examiners were stored on an unidentified hard drive [18].

#### 2.4.1. Diaphragmatic Assessment

Ultrasonography is an instrument that allows the evaluation of diaphragmatic thickness and mobility. For this study, the diaphragmatic thickness was evaluated. A high-frequency linear array transducer was used to measure the thickness of the diaphragm using B-mode. The transducer was positioned in the 8th or 9th intercostal space between the middle and anterior axillary lines in the zone of apposition. The diaphragm was visualized as the intermediate space between the pleural and peritoneal lines at the end of expiration [24]. Even though it is known that there are some influences of the mechanical ventilator’s parameters on the diaphragm thickness, participants with mechanical ventilation were assessed by all examiners under the same circumstances and parameters.

#### 2.4.2. Peripheral Muscles Assessment

Peripheral muscles (biceps brachii, forearm flexors, quadriceps femoris and tibialis anterior) were assessed through the protocol described by Arts et al. [25] and Gruther et al. [26]. All participants were examined in the supine position, with arms and legs extended and muscles in a relaxed state. The evaluation was performed in the transverse plane, in the largest diameter of the analyzed muscle, and with the amount of contact gel necessary to minimize the pressure of the transducer on the skin [25,26].

**Figure 1:**
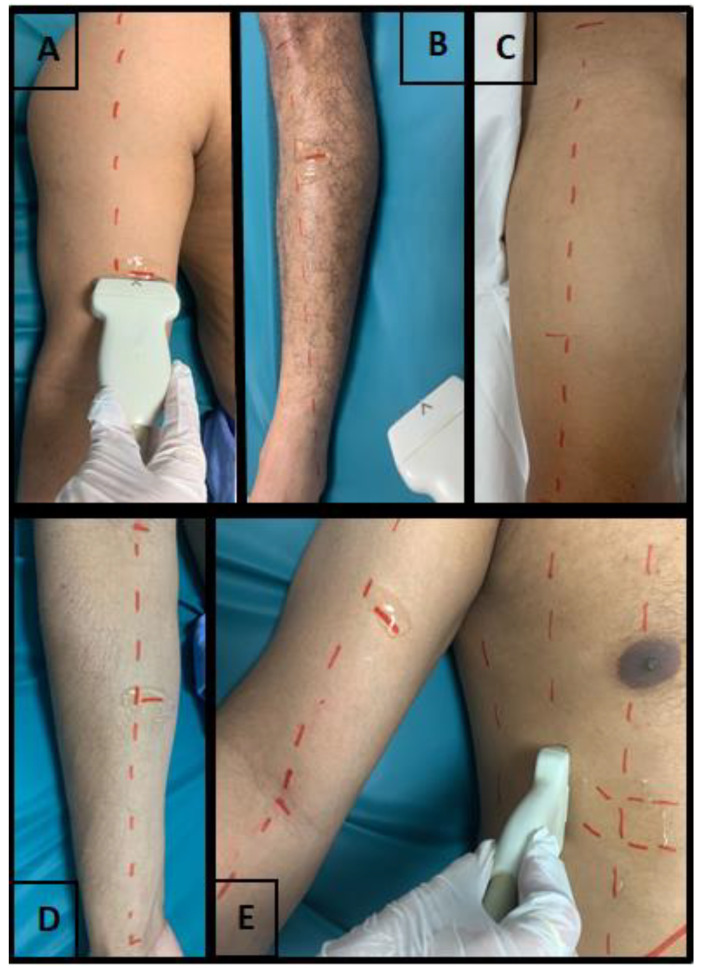
US transducer positioning during assessment. Source: authors. (**A**): Biceps Brachii; (**B**): Tibialis anterior; (**C**): Quadriceps femoris; (**D**): Forearm flexor groups; (**E**): Diaphragm.

To obtain images of the biceps brachii, it was positioned the transducer perpendicularly along the axis of the arm on its anterior surface at the point located between the lower third and the upper two-thirds of the distance between the acromion and the cubital fossa. For the forearm flexor group, the transducer was positioned between the upper two-fifths and the lower three-fifths of the distance from the antecubital crease to the distal end of the radius. For the quadriceps femoris, the transducer was positioned perpendicularly to the muscle at the point located two-thirds of the distance between the anterior superior iliac spine and the superior surface of the patella on the anterior thigh. For the tibialis anterior muscle, the transducer was positioned perpendicularly to the long axis of the muscle at the point between the upper quarter and lower three-quarters of the distance from the lower border of the patella to the tip of the lateral malleolus [25,26]. Approximate transducer position can be seen in Figure 1.

#### 2.4.3. Muscle Thickness

**Figure 2:**
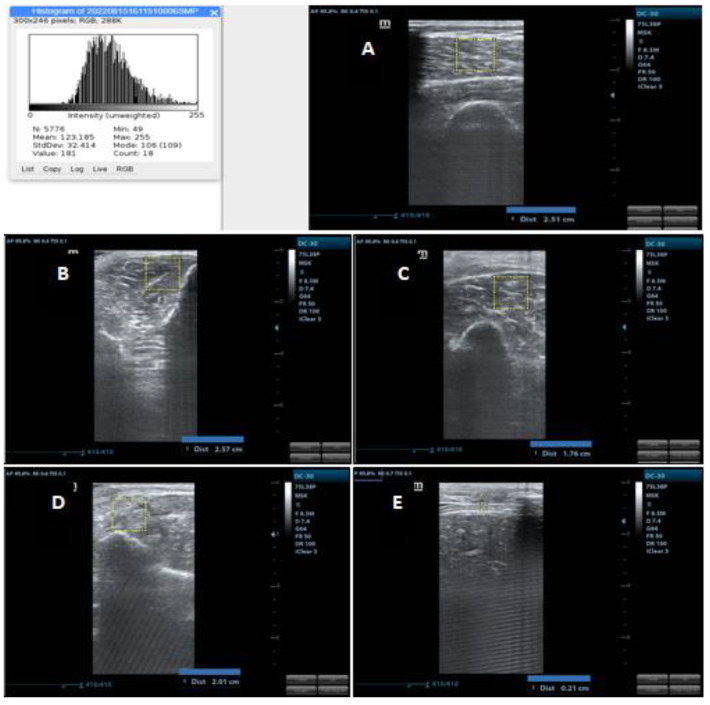
US image demonstrating various measurements of muscle thickness and echogenicity. Source: authors. (**A**) Quadriceps femoris US image with thickness measurement and histogram; (**B**) Tibialis anterior US image with thickness measurement; (**C**) Biceps Brachii US image with thickness measurement; (**D**) Forearm flexor groups US image with thickness measurement; (**E**) Diaphragm US image with thickness measurement.

The thickness of the biceps brachii muscle was assessed between the upper part of the humerus and the superficial fascia of the biceps (including the brachialis muscle); on the forearm flexor group, it was evaluated between the interosseous membrane located close to the radius and the superficial fascia of the flexors; on the quadriceps femoris it was evaluated between the upper part of the femur and the superficial fascia of the rectus femoris (includes the rectus femoris and vastus intermedius); finally, on the tibialis anterior it was evaluated between the interosseous membrane located close to the tibia and the superficial fascia of the tibialis anterior [25]. Measurements were performed in centimeters and can be seen in Figure 2.

#### 2.4.4. Muscle Echogenicity

Muscle echogenicity was quantified using the grayscale analysis histogram through the square trace method using the ImageJ software in a region of interest with an area of 2 cm × 2 cm for the peripheral muscles and 1 cm × 1 cm for the diaphragm. In muscles where the square was not allowed in the indicated measurements, the largest possible square area was examined within the anatomical limits established for the evaluated muscles. This area was used to reduce the risk of using structures other than the muscle of interest, such as fascia and bone. In grayscale analysis, black is assigned 0, and white is assigned 255, with shades of gray in between and each pixel is assigned a value. This allows the mean and standard deviation of grayscale values to be calculated, which was repeated 3 times for each muscle [27]. Images used for echogenicity analyses were selected from those used for the assessment of muscle thickness, using only the images of one examiner, which was randomly chosen. Measurements can be seen in Figure 1. As the training did not include the echogenicity assessment, only the examiner who had experience with the assessment and use of ImageJ carried out the analysis, so only the intra-examiner reliability was performed.

### 2.5. Statistical Analysis

For descriptive analyses, categorical variables were expressed in frequency (percentage), and continuous variables were submitted to Shapiro–Wilk test to determine the normality of the distribution. The variables met the normality criteria and were presented as mean and standard deviation. For the inter and intraobserver reliability analyses, the values of the Intraclass Correlation Coefficient (ICC) were performed, considering a two-way mixed-effects method with absolute agreement. Regarding thickness, the mean thickness measurement of the three images captured was performed for the inter-examiner reliability analysis for each muscle. For intra-examiner reliability, the analysis was performed based on the measurements of the three images of each evaluator for each muscle. In regards to echogenicity, only one evaluator carried out the analyses, so only the intraobserver reliability was performed, using the average value of echogenicity obtained in ImageJ, as previously reported. A weak correlation was considered when a value lower than 0.4 was found; satisfactory correlation with a value greater than or equal to 0.4 and less than 0.60; a value equal to or greater than 0.60 and less than 0.75 the correlation was considered good; a value greater than or equal to 0.75 the correlation was considered excellent [28]. All analyzes were performed using SPSS (v.20.0, IBM), considering a significance level of *p* ≤ 0.05 and a 95% confidence interval (CI).

## 3. Results

The study sample was 10 participants, which resulted in the acquisition and analysis of 600 US images for inter and intra-examiner muscle thickness reliability and 150 US images for intra-examiner muscle echogenicity reliability. The sample presented a mean age of 62 years old, most of whom were male (60%), with a mean stay of 9.1 days in the ICU on the day of the US assessment. In regards to health status, the majority had a clinical condition upon admission to the ICU (70%), used mechanical ventilation during their ICU stay (90%), and had SAPSII with a mean of 68.4. Participants’ sociodemographics information can be seen in Table 1.

The values of muscle thickness and echogenicity were presented as means and standard deviations, shown in Table 2 and Table 3, respectively. In regards to muscle thickness, the averages of the three assessments performed on each muscle group per examiner were presented. For muscle echogenicity, the averages of three assessments performed for each muscle group by the single examiner were arranged.

In regards to muscle echogenicity, only intra-examiner reliability was performed. Excellent results were found in all analyses performed (ICC: 0.867–0.973). The results can be seen in Table 3.

In regards to muscle thickness, inter-rater and intra-rater reliability analyses were performed. For inter-examiner reliability, excellent results were found for all muscle groups (ICC: 0.778–0.942). As for the intra-examiner reliability, excellent results were found in the vast majority of analyses performed (ICC: 0.718–0.988), with only a “good” correlation in the intra-examiner reliability of examiner 1 of the diaphragm muscle (ICC: 0.718). The results can be seen in Table 4.

## 4. Discussion

Considering that this study aimed to analyze the inter-examiner and intra-examiner reliability of the ultrasonography assessment of muscle thickness and echogenicity of peripheral and respiratory muscles, the results demonstrated excellent inter- and intra-examiner reliability of muscle thickness assessment and intra-examiner echogenicity assessment of biceps brachii, forearm flexors groups, quadriceps femoris, tibialis anterior and diaphragm of critically ill patients.

Firstly, it is important to highlight that this study makes an important advance in scientific knowledge with the implementation of a training/qualification program for health professionals with different backgrounds. Studies have requested that training programs should be structured to enhance the reliability of US muscle assessment, as there is still no training program or standardized protocols for training health professionals and students [8,19,20,29,30]. In addition, it is also suggested that studies should report data regarding reliability and training, not considering that these are already established in the literature [20].

The sample of this study corresponds, for the most part, to males and older adults, with a high risk of mortality who used MV during hospitalization and with a long period of hospitalization, which may have influenced the results found. These factors may explain the reduced muscle thickness and the poorer muscle quality evidenced by echogenicity when compared to healthy individuals and critically ill subjects from other studies. These conditions make these assessments even more challenging, increasing the importance of the results presented in this study.

The assessment, as well as the reliability, of muscle thickness, is the most widespread US muscle analysis technique in the literature. As expected, it is clear that small variations of muscle measurements can occur due to various reasons, as shown in Table 2. For this reason, it is suggested to use the mean value of three measurements, as was done [7]. However, when ICC was performed, the excellent correlation between examiners was demonstrated, which took into consideration the absolute agreement between examiners; in other words, it considered both the agreements and systematic differences between measurements [31]. This study corroborates those studies carried out in ICUs, demonstrating excellent inter- and intra-examiner reliability of examiners with little or no experience in muscle assessment via US [17,18,32].

Considering the muscles evaluated in this study, the quadriceps femoris is the muscle most analyzed in studies [5,17,18,33], probably because it has large dimensions and well-defined anatomical conditions, a fact that can make its assessment easier [17]. This study corroborates previous studies that demonstrated excellent inter- and intra-examiner reliability in assessing the quadriceps femoris in the ICU and with examiners with different levels of experience [5,17,18,33].

In association with the quadriceps femoris, some studies also analyzed the inter- and intra-examiner reliability of the Tibialis anterior thickness assessment [18,33]. Comparing the results between experienced and inexperienced examiners with different professional backgrounds, Mayer et al. [18] found excellent inter-examiner (ICC: 0.886) and intra-examiner (ICC: 0.761–0.857) reliability for Tibialis anterior thickness assessment, as well as quadriceps femoris (ICC: 0.915 and 0.884–0.901) and biceps brachii (ICC: 0.958 and 0.924–0.962) [18].

In addition, the inter- and intra-examiner reliability of the diaphragm thickness assessment is also highly evaluated [17,34,35]. Corroborating with the findings of Sarwal et al. [17], results showed less agreement between the evaluators, despite the result being considered “excellent”, with only one intra-examiner reliability considered “good” for one of the examiners. This result can be explained by the difficulty in identifying and analyzing this muscle. The diaphragm is a thin muscle with an average of 0.33 cm in healthy individuals [36]. In the study by Sarwal et al. [17], diaphragmatic thickness averaged between 0.22 cm and 0.26 cm, and in our study, 0.20 cm and 0.22 cm, varying between examiners.

Finally, the muscles of the upper limb are less analyzed in relation to the quadriceps femoris. Our results corroborate the findings in the literature for the biceps brachii [18,32,33] and forearm flexors group [37]. There was less agreement, despite the excellent result, for interobserver reliability in the forearm flexor group. This result can be explained by the need for greater positioning adjustments due to range of motion limitations for forearm supination found in the participants, which are due to muscle atrophy and edema in the assessed region.

Regarding echogenicity, reliability studies are scarce in the literature, which does not reflect its importance for the diagnosis of acquired muscle weakness and reduced functional capacity during hospitalization and discharge from the ICU [11,12,13]. This study results demonstrated excellent intra-examiner reliability for all analyzed muscle groups, corroborating previously published results in relation to the quadriceps femoris [17,18,38,39], tibialis anterior [18], biceps brachii [18] and diaphragm [17]. There were not found studies analyzing the reliability of echogenicity in the forearm flexors group in critically ill patients.

Our study had as strengths the presentation of a training program for health professionals from different areas of training, which allows the expansion of the use of US for muscle assessment. As stated in many prior studies, minimal training is required to perform US muscle assessment, with a study giving a 20 min training for that [40]. This study established an 8 h practical training, and a “guiding document” was available during the image acquisition, including the step-by-step to be performed to acquire and analyze the US images. So, it should be clear that, after the training, these professionals were able to perform image acquisition and analysis with an excellent analysis correlation. Furthermore, this study expanded the possibilities of using a higher number and less common muscle groups through US assessment of muscle thickness and echogenicity.

As limitations, there was no inclusion of radiologists in the team, which may facilitate the handling and training for US muscle assessment. However, studies have shown excellent reliability in examiners with different levels of experience and professional training [17,18,32]. Moreover, as this research was performed in a single center as well as included small sample size, it may be difficult to generalize the results to other populations.

## 5. Conclusions

Considering all the aspects presented, it was possible to demonstrate excellent inter- and intra-examiner reliability in the US assessment of muscle thickness and intra-examiner muscle echogenicity of the biceps brachii, forearm flexors, quadriceps femoris, tibialis anterior and diaphragm of individuals admitted to the ICU, through a training program for inexperienced professionals with different professional backgrounds.

The protocol presented in this study, as well as its results, might support the clinical practice of ICU professionals, in addition to favoring the development of scientific studies with the application of ultrasonography in the quantitative and qualitative assessment of the peripheral and respiratory muscles. However, it is recommended that future studies should include an expert in US muscle assessment in the data collection team as a standard of comparison, as well as larger sample sizes to improve the results generalization.

## Figures and Tables

**Table 1:** Participants’ Sociodemographic and clinical characteristics.

Characteristics	Results
Age in years (Mean ± SD)	62 (±18.92)
Sex	
Male (%)	60%
Female (%)	40%
Condition for admission to the ICU	
Clinical (%)	70%
Surgical (%)	30%
Days of ICU stay at the time of assessment (Mean ± SD)	9.10 (±6.14)
Use of mechanical ventilation during ICU stay	
Yes (%)	90%
No (%)	10%
SAPSII (Mean ± SD)	68.40 (±16.10)

**Table 2:** Mean and standard deviation of muscle thickness per examiner.

	Examiners	Mean (±SD) in cm
Biceps brachii	Examiner 1	2.47 (±0.70)
	Examiner 2	1.98 (±0.52)
	Examiner 3	2.46 (±0.52)
	Examiner 4	2.59 (±0.61)
Forearm flexor group	Examiner 1	2.46 (±0.47)
	Examiner 2	2.01 (±0.34)
	Examiner 3	2.53 (±0.52)
	Examiner 4	2.32 (±0.40)
Quadriceps femoris	Examiner 1	2.64 (±0.64)
	Examiner 2	2.68 (±0.83)
	Examiner 3	3.06 (±0.87)
	Examiner 4	2.73 (±0.70)
Tibialis anterior	Examiner 1	1.95 (±0.29)
	Examiner 2	1.92 (±0.28)
	Examiner 3	2.02 (±0.28)
	Examiner 4	1.96 (±0.24)
Diaphragm	Examiner 1	0.20 (±0.06)
	Examiner 2	0.21 (±0.08)
	Examiner 3	0.22 (±0.07)
	Examiner 4	0.21 (±0.04)

**Table 3:** Mean, standard deviation and intra-examiner reliability of muscle echogenicity.

	Biceps Brachii	Forearm Flexor Group	Quadriceps Femoris	Tibialis Anterior	Diaphragm
Mean (±SD) in cm	119.23 (±27.15)	131.30 (±27.56)	124.65 (±28.37)	137.17 (±22.71)	124.43 (±24.83)
ICC	0.952	0.973	0.970	0.954	0.867
Correlation	Excellent	Excellent	Excellent	Excellent	Excellent

**Table 4:** Intra-examiner and inter-examiner reliability of muscle thickness according to muscle group.

Muscles	Intra-Examiner Reliability			Inter-Examiner Reliability	
	Examiners	Results	Correlation	Results	Correlation
Biceps brachii	Examiner 1	0.982	Excellent	0.894	Excellent
	Examiner 2	0.951	Excellent		
	Examiner 3	0.977	Excellent		
	Examiner 4	0.988	Excellent		
Forearm flexor group	Examiner 1	0.972	Excellent	0.789	Excellent
	Examiner 2	0.918	Excellent		
	Examiner 3	0.942	Excellent		
	Examiner 4	0.922	Excellent		
Quadriceps femoris	Examiner 1	0.950	Excellent	0.917	Excellent
	Examiner 2	0.984	Excellent		
	Examiner 3	0.972	Excellent		
	Examiner 4	0.960	Excellent		
Tibialis anterior	Examiner 1	0.924	Excellent	0.942	Excellent
	Examiner 2	0.913	Excellent		
	Examiner 3	0.930	Excellent		
	Examiner 4	0.902	Excellent		
Diaphragm	Examiner 1	0.718	Good	0.778	Excellent
	Examiner 2	0.972	Excellent		
	Examiner 3	0.931	Excellent		
	Examiner 4	0.798	Excellent		

## Data Availability

The data presented in this study are available upon reasonable request from the corresponding author.
